# The B6 database: a tool for the description and classification of vitamin B6-dependent enzymatic activities and of the corresponding protein families

**DOI:** 10.1186/1471-2105-10-273

**Published:** 2009-09-01

**Authors:** Riccardo Percudani, Alessio Peracchi

**Affiliations:** 1 Department of Biochemistry and Molecular Biology, University of Parma, 43100 Parma, Italy

## Abstract

**Background -:**

Enzymes that depend on vitamin B6 (and in particular on its metabolically active form, pyridoxal 5'-phosphate, PLP) are of great relevance to biology and medicine, as they catalyze a wide variety of biochemical reactions mainly involving amino acid substrates. Although PLP-dependent enzymes belong to a small number of independent evolutionary lineages, they encompass more than 160 distinct catalytic functions, thus representing a striking example of divergent evolution. The importance and remarkable versatility of these enzymes, as well as the difficulties in their functional classification, create a need for an integrated source of information about them.

**Description -:**

The B6 database  contains documented B6-dependent activities and the relevant protein families, defined as monophyletic groups of sequences possessing the same enzymatic function. One or more families were associated to each of 121 PLP-dependent activities with known sequences. Hidden Markov models (HMMs) were built from family alignments and incorporated in the database. These HMMs can be used for the functional classification of PLP-dependent enzymes in genomic sets of predicted protein sequences. An example of such analyses (a census of human genes coding for PLP-dependent enzymes) is provided here, whereas many more are accessible through the database itself.

**Conclusion -:**

The B6 database is a curated repository of biochemical and molecular information about an important group of enzymes. This information is logically organized and available for computational analyses, providing a key resource for the identification, classification and comparative analysis of B6-dependent enzymes.

## Background

The term 'vitamin B6' refers to a collective of six biologically interconvertible 3-hydroxy-2-methylpyridine compounds: pyridoxal, pyridoxine, pyridoxamine, and their respective 5'-phosphates. Among these, pyridoxal 5'-phosphate (PLP) is the main metabolically active form, serving as a cofactor for a variety of enzymes in all organisms [1-7].

**Figure 1:**
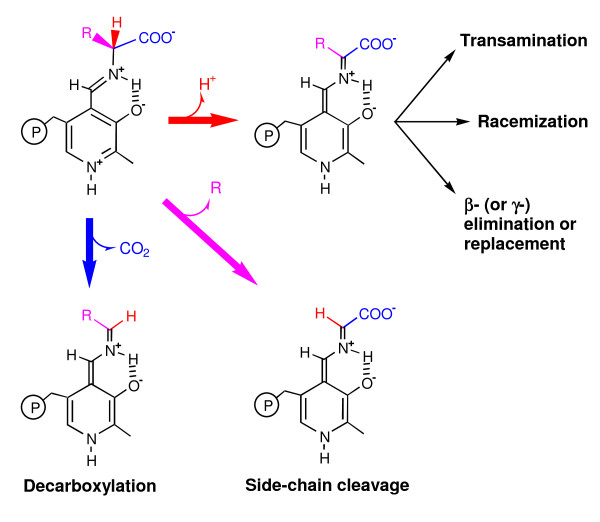
**A schematic view of the different reaction types catalyzed by PLP-dependent enzymes that act on amino acids**. In these enzymes, PLP is bound to the ε-amino group of a catalytic lysine residue, forming a Schiff base (internal aldimine). Covalent binding of the substrate amino acid occurs through a transimination reaction, leading to formation of an external aldimine intermediate (structure on the upper left corner). Subsequently, the protonated ring system of PLP acts as an electron sink, to stabilize species carrying a negative charge on the α-carbon (carbanions). Depending on the enzyme (and hence on the specific arrangement of the active site residues) such stabilized carbanions can be formed upon cleavage of any of the three covalent bonds connecting the α-carbon to its substituents. Removal of the carboxylate group is typical of decarboxylases. Removal of the amino acid side chain occurs for example in threonine aldolase. Finally, removal of the α-proton may be the prequel to the formation of various further intermediates, leading to racemization, cyclization, β- and γ-elimination, and transamination reactions [1,4,7].

Nearly all PLP-dependent enzymes, with the exception of glycogen phosphorylases, are associated with biochemical pathways involving amino compounds - mostly amino acids. The reactions catalyzed by the PLP-dependent enzymes that act on amino acids include transamination, decarboxylation, racemization, and eliminations or replacements at the β- or γ-carbons. Such versatility arises from the fact that PLP can covalently bind the substrate and then act as an electrophilic catalyst, stabilizing different types of carbanionic reaction intermediates [7] (Figure 1).

The Enzyme Commission (EC; ) lists more than 140 PLP-dependent activities, corresponding to ~4% of all classified activities [6]. Despite this wide functional variety, all structurally characterized PLP-dependent enzymes have been classified into just five distinct structural groups (also known as 'fold types') [4,8], which presumably correspond to independent evolutionary lineages [3,5]. This represents a remarkable example of divergent evolution, meaning that proteins with similar structure and sequence can perform different chemical reactions. Due to the mechanistic similarities between PLP-dependent enzymes and to their limited structural diversity, inferring the function of these catalysts solely based on sequence similarity entails particular difficulties.

To help the identification and classification of sequences belonging to PLP-dependent enzymes, we have created the B6 database. In addition to a wealth of links to other Internet resources (including BRENDA [9] and the PLP mutant enzyme database[10]), the B6 database contains over 180 documented PLP-dependent activities that are associated, when possible, to one or more protein families (defined as monophyletic groups of homologous proteins sharing the same function). The database also contains hidden Markov models (HMMs) that were built from family alignments and that can be employed for the identification and functional classification of PLP-dependent enzymes in genomic sets of protein sequences. Indeed, we have used these HMMs to scan a series of complete genomes, obtaining a census of predicted PLP-dependent enzymes in various organisms.

## Construction and content

### Organization and statistics of the B6 database

**Figure 2:**
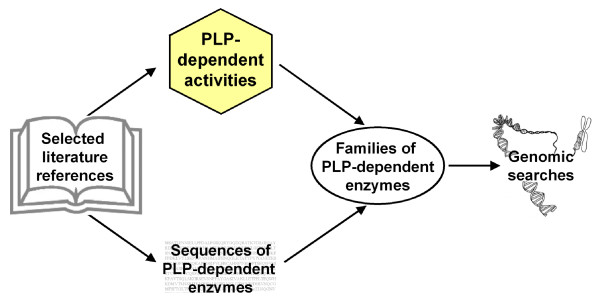
**The B6 database relational structure**. This figure delineates the four modules composing the database and their relationships. The database of PLP-dependent enzyme families was assembled based on the examination of the literature and on a collection of functionally validated sequences, as described in the text. These families, and in particular the HMMs associated to them, have been used for the identification and functional classification of PLP-dependent enzymes in sets of predicted protein sequences from whole genomes.

Figure 2 summarizes the structure of the database, illustrating the types of information it includes and the ways in which this information is linked together and can be searched. As shown, the B6 database site actually accesses and integrates four distinct databases, namely a list of PLP-dependent activities, a collection of pertinent literature references, a large set of sequences of PLP-dependent proteins (grouped into protein families) and the results of our genomic searches.

The B6 database release 1.0 (as of 15/05/2009) includes 184 activities and over 2000 sequences of B6-dependent enzymes, subdivided into 149 families. For each family, the database provides a multiple sequence alignment and the derived hidden Markov model.

### Assembly of the databases: activities, sequences and protein families

The B6 database was constructed based on an inventory of documented B6-dependent activities, most but not all of which have been catalogued by the Enzyme Commission and are therefore associated to an official EC number. A systematic examination of the literature showed that 121 of these activities could be associated to enzymes of known sequences, and in these cases we proceeded to the creation of protein families, that we define as monophyletic groups of sequences all possessing the same enzymatic activity. Each given activity was associated to one or more families based on this criterion.

The number of sequences in individual families was then increased by homology searches, i.e. by scanning the GenBank with BLAST [11] or with psi-BLAST [12], using as query the functionally validated protein(s). Criteria for inclusion of a sequence in a family were the following:

(1) Only sequences yielding an E value < 10^-10 ^were generally considered (this limit could be somewhat lowered for families composed of short sequences).

(2) Sequences showing a >90% identity to a protein of known function were usually not included, to diminish redundancy.

(3) Sequences being substantially (>30%) shorter than the shortest functionally validated sequence in the family were discarded. Sequences lacking the PLP-binding lysine residue were also discarded (except for rare cases in which the protein is known not to bind PLP via a lysine).

(4) Sequences showing a higher similarity to other characterized PLP-dependent enzymes (i.e., to some functionally validated protein belonging to another family) were discarded.

(5) Finally, sequences from taxa in which the enzymatic activity of the family was not documented, were also generally discarded.

Multiple alignments were constructed with ClustalW [13]. Given that the families were composed of closely related sequences, these alignments did not need to be manually adjusted or to be guided by structural information (even when available).

The ProDom program [14] was used for alignment inspection and phylogenetic analysis. Family alignments were used to build Hidden Markov Models (HMM) with programs of the HMMER suite [15]. The scores of sequences included or excluded from a given family were then calculated with respect to the family HMM. From this procedure, score cut-offs for each family were determined and then used for sequence classification.

A family HMM is a probabilistic model, constructed from a multiple alignment, which describes the sequence conservation within a protein family. In comparison to consensus sequences or similar regular expressions, HMMs provide a more articulated modeling of the features of a protein family. Such higher complexity is responsible for the greater discriminatory power of the HMM methodology in the identification of other putative family members [15]. Depending on family inclusion criteria and score thresholds, HMMs can be used to identify homology at different levels of granularity. The 'family' definition adopted in the B6 database is similar to the 'equivalog family' definition of TIGRFAM [16], while a single family in PFAM [17] typically corresponds to many different families in our database.

### Cluster analysis of PLP-dependent enzyme families

**Figure 3:**
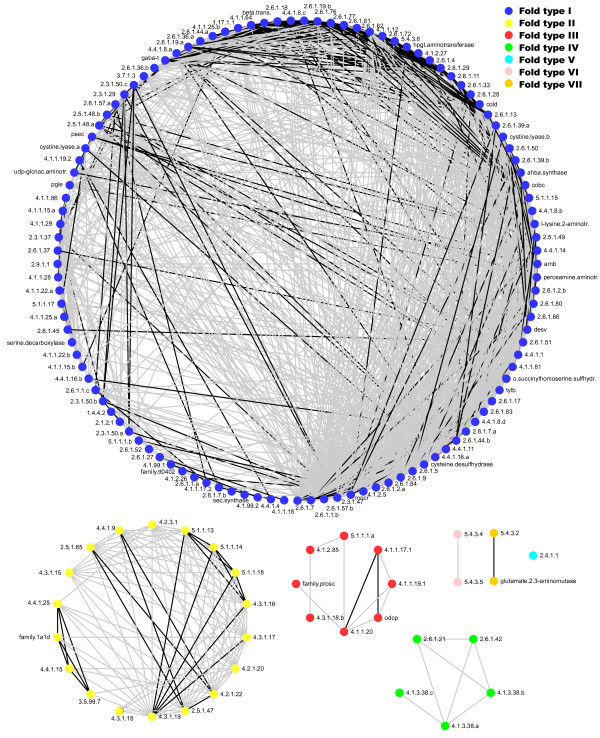
**Homology network of PLP-dependent enzymes**. Nodes represent Hidden Markov models (HMMs) of PLP-dependent families. Edges represent homology connections (E < 10^-5^) between families established by HMM-HMM comparisons [18]. Black edges connect protein families with the most significant similarities (E < 10^-50^). The network is visualized with the "Degree sorted circle layout" of Cytoscape [19]. Colors were mapped into nodes using the structural group of the protein family as a node property.

To elucidate the relationships between the 149 enzyme families defined as above, we performed an all *versus *all comparison of the families in the database using an HMM-HMM alignment software [18]. The results of this comparison were analyzed with an interaction network software [19] to build an homology-based network of PLP-dependent families (Figure 3). By considering only significant similarities (E < 10^-5^) between HMMs, the analysis identified seven separated clusters of PLP-dependent families (Figure 3). Five of these clusters corresponded to the traditional classification of PLP-dependent enzymes into five distinct structural groups (fold types I to V). Of the two additional clusters, one included lysine 5,6-aminomutase (EC: 5.4.3.4) and the other lysine 2,3-aminomutase (EC: 5.4.3.2) - two enzymes whose structures have been recently determined and found to be different from the known structures of PLP-dependent enzymes [20,21]. In the database, the protein families belonging to these two clusters were assigned, respectively, to fold types VI and VII.

Since HMM-HMM comparison is very sensitive to sequence similarity, it can reveal faint evolutionary relationships between protein families. This information can be particularly useful to identify relatives for PLP-dependent families that fail to reveal similarity with other families when analyzed by sequence-sequence (e.g., Blast) or sequence-HMM (e.g. HMMPFAM) methods. The HMM-HMM analysis, for example, indicates a significant similarity between Prosc (a family of proteins with unknown function) and diaminopimelate decarboxylase (EC: 4.1.1.20) - a relationship that is not apparent through Blast or HMMPFAM comparisons.

Inter-families distances deriving from HMM-HMM comparisons served as a guide to build alignments representative of the seven distinct structural groups. Distance matrices among families were analyzed with an UPGMA algorithm and a rapid multiple sequence alignment method [22] was used to progressively align PLP-dependent families belonging to the same structural type. From these alignments, we constructed HMMs (hereafter named "fold-type HMMs") representative of the seven structural groups of PLP-dependent enzymes.

## Utility and discussion

The B6 database is a repository in which detailed (biochemical and genetic) information about an important group of enzymes is concentrated, organized and made available for computational analyses. We expect that the B6 database will be a valuable tool for experimental researchers in the PLP field, but also a reference point for the design of theoretical studies by bioinformaticians.

In particular, the sequence information accumulated in the database can be used to facilitate the identification and functional assignment of B6-dependent enzymes. To illustrate this point, we employed the family and fold-type HMMs (constructed as described above) to search and preliminarily classify PLP-dependent enzymes in genomic sets of predicted proteins. The results of such analyses have also been incorporated in the database.

Complete sets of protein sequences deduced from genomic data were generally obtained from NCBI  or from similar ftp repositories. The classification of protein sequences was achieved through a two-step procedure. First, each sequence was compared with our database of PLP-dependent sequences by performing a HMM search with the seven fold-type HMMs, using relaxed significance criteria (E ≤ 10^-1^; database size = 10000). This step served as a quick filter to sift out genes that were likely to code for PLP-dependent enzymes. Candidates were subsequently compared with the library of family HMMs using HMMPFAM. This step was more time-consuming and served for a preliminary functional classification of the proteins.

A protein was considered to possess the same activity as its best-hit family if it exhibited a significant similarity to the family HMM (E ≤ 10^-3^) and a score above a 'trusted' cut-off established by the family curator. Sequences with a score below this threshold were marked as 'low-score' to indicate their modest similarity to the family model. These sequences were not considered as possessing the enzymatic function of the family, but were regarded as possessing an uncharacterized, possibly related, activity. According to this analysis, very few sequences exhibited a significant similarity to a fold-type HMM (E ≤ 10^-3^) but no significant similarity to any family HMMs. In such cases, sequences were considered as potential PLP-dependent enzymes with an uncharacterized catalytic activity.

To further characterize the protein sequence under examination, the classification program searched for a putative PLP-binding lysine residue (see legend of Figure 1). This was achieved by aligning the sequence with validated family members in which the position of the catalytic lysine had been previously mapped. This analysis can reveal proteins that are evolutionary related to PLP-dependent enzymes, but have lost the ability to bind the PLP cofactor.

### Example: a census of human genes that encode PLP-dependent enzymes

By employing the approach outlined above, we searched the latest draft of the human genome (NCBI 36 assembly, downloaded at ) to obtain an inventory of the human genes coding for PLP-dependent enzymes. The initial output of the program (69 sequences recognized as probable PLP-dependent proteins) was further analyzed to identify pseudogenes, false positives and entries representing alternative protein isoforms.

The search identified 56 expressed genes coding for PLP-dependent proteins (Table 1. Note that the products of genes SPTLC1, ADC and AZIN1, albeit homologs of *bona fide *PLP-dependent enzymes, appear to have acquired a nonenzymic function during evolution). Thirteen more proteins were recognized as isoforms deriving from some of the genes above. To appreciate the rate of false negatives in our analysis, we performed an extensive text search in the GenBank database of human genes, to identify all those genes annotated (directly or indirectly) to code for B6-dependent proteins. However, we found no hits other than the 56 genes listed in Table 1, which therefore represent, to the best of our current knowledge, the complement of human PLP-dependent genes.

**Table 1:** Inventory of the human genes that encode PLP-dependent enzymes

**Activity**	**Family**	**Protein accession #**	**E-value**	**Isoforms**	**Gene**	**Chromosome**
Glycine dehydrogenase	1.4.4.2	NP_000161	0		GLDC	9
Glycine hydroxymethyltransferase	2.1.2.1	NP_004160	2 e-294	NP_683718	SHMT1	17
Glycine hydroxymethyltransferase	2.1.2.1	NP_005403	8 e-289		SHMT2	12
Glycine C-acetyltransferase	2.3.1.29	NP_055106	4 e-225		GCAT	22
5-aminolevulinic acid synthase	2.3.1.37	NP_954635	1 e-269	NP_000679	ALAS1	3
5-aminolevulinic acid synthase	2.3.1.37	NP_000023	2 e-270	NP_001033057 NP_001033058 NP_001033056	ALAS2	X
Serine C-palmitoyltransferase	2.3.1.50.a	NP_006406	6 e-240		SPTLC1^(a)^	14
Serine C-palmitoyltransferase	2.3.1.50.b	NP_004854	6 e-299		SPTLC2	20
Serine C-palmitoyltransferase	2.3.1.50.b	NP_060797	3 e-260		SPTLC3	20
Phosphorylase	2.4.1.1	NP_002854	0		PYGL	14
Phosphorylase	2.4.1.1	NP_005600	0		PYGM	11
Phosphorylase	2.4.1.1	NP_002853	0		PYGB	20
Aspartate aminotransferase	2.6.1.1.a	NP_002070	2 e-295		GOT1	10
Aspartate aminotransferase	2.6.1.1.a	NP_689626	1 e-68		GOT1L1	8
Aspartate aminotransferase	2.6.1.1.a	NP_002071	2 e-306		GOT2	16
Alanine aminotransferase	2.6.1.2.b	NP_005300	6 e-262		GPT	8
Alanine aminotransferase	2.6.1.2.b	NP_597700	3 e-267		GPT2	16
Tyrosine aminotransferase	2.6.1.5	NP_000344	2 e-292		TAT	16
Kynurenine:oxoglutarate aminotransf.	2.6.1.7	NP_004050	7 e-279		CCBL1	9
Kynurenine:oxoglutarate aminotransf.	2.6.1.7	NP_001008662	4 e-266	NP_001008661	CCBL2	1
Ornithine:oxo-acid aminotransferase	2.6.1.13	NP_000265	4 e-274		OAT	10
4-aminobutyrate aminotransferase	2.6.1.19.a	NP_065737	0	NP_000654	ABAT	16
2-aminoadipate aminotransferase	2.6.1.39.a	NP_872603	3 e-265	NP_057312	AADAT	4
Branched-chain aa aminotransferase	2.6.1.42	NP_005495	3 e-191		BCAT1	12
Branched-chain aa aminotransferase	2.6.1.42	NP_001181	1 e-181		BCAT2	19
Alanine:glyoxylate aminotransferase	2.6.1.44.a	NP_114106	0		AGXT2	5
Alanine:glyoxylate aminotransferase	2.6.1.44.a	NP_112569	1 e-114		AGXT2L1^(b)^	4
Alanine:glyoxylate aminotransferase	2.6.1.44.a	NP_699204	8 e-100		AGXT2L2^(b)^	5
Serine:pyruvate aminotransferase	2.6.1.51	NP_000021	7 e-269		AGXT	2
Phosphoserine aminotransferase	2.6.1.52	NP_478059	8 e-235	NP_066977	PSAT1	9
Cysteine desulfurase	2.8.1.7.a	NP_066923	5 e-290		NFS1	20
Cysteine desulfurase	2.8.1.7.b	NP_060417	0		MOCOS	18
Kynureninase	3.7.1.3	NP_003928	3 e-259		KYNU	2
Glutamate decarboxylase	4.1.1.15.a	NP_000808	0		GAD1	2
Glutamate decarboxylase	4.1.1.15.a	NP_000809	0		GAD2	10
Ornithine decarboxylase	4.1.1.17.1	NP_002530	1 e-175		ODC1	2
Histidine decarboxylase	4.1.1.22.b	NP_002103	1 e-285		HDC	15
Aromatic-L-amino-acid decarboxylase	4.1.1.28	NP_000781	2 e-291	NP_001076440	DDC	7
Sulfinoalanine decarboxylase	4.1.1.29	NP_997242	2 e-116		GADL1	3
Sulfinoalanine decarboxylase	4.1.1.29	NP_057073	0		CSAD	12
Sphinganine-1-phosphate aldolase	4.1.2.27	NP_003892	9 e-249		SGPL1	10
Cystathionine beta-synthase	4.2.1.22	NP_000062	2 e-228		CBS	21
Threonine synthase	4.2.3.1	NP_079114	7 e-91		THNSL1	10
Threonine synthase	4.2.3.1	NP_060741	4 e-80		THNSL2^(c)^	2
L-serine ammonia-lyase	4.3.1.17	NP_006834	5 e-209		SDS	12
L-serine ammonia-lyase	4.3.1.17	NP_612441	2 e-205		SDSL	12
Cystathionine gamma-lyase	4.4.1.1	NP_001893	7 e-296	NP_714964	CTH	1
1-ACC synthase	4.4.1.14	NP_115981	1 e-81		ACCS^(d)^	11
1-ACC synthase	4.4.1.14	NP_001027025	3 e-78		ACCSL	11
Selenocysteine lyase	4.4.1.16.b	NP_057594	0		SCLY	2
Serine racemase	5.1.1.18	NP_068766	8 e-243		SRR	17
Ornithine decarboxylase paralogue	odcp	NP_443724	8 e-197		ADC^(e)^	1
Ornithine decarboxylase paralogue	odcp	NP_056962	2 e-116	NP_680479	AZIN1^(e)^	8
L-phosphoseryl-tRNA^Ser^:seleno- phosphate seleniumtransferase	sec.synthase	NP_722547	0	NP_058651	SEPSECS	4
Unclassified activity	Uncharact. family.prosc	NP_009129	5 e-166		PROSC	8
Unassigned	n/a	NP_055842	9 e-8		PDXDC1	16

The complete set of proteins from the NCBI 36 assembly of the human genome was searched as described in the text. The 'Family' column reports the name of the family whose HMM scored best when compared to the query. When possible, the family name is constituted by, or includes, the four-digits EC number of the corresponding activity. Note that the layout of the present table recapitulates the output provided by B6 database's site, with some modifications. In particular, the occurrence of protein isoforms derived from some genes was obtained by analysis of the crude program results. The official names of the genes corresponding to the transcripts and their chromosomal locations were obtained from NCBI.

(a) This gene encodes the subunit LCB1 of human serine palmitoyltransferase. This subunit is not itself a PLP-dependent enzyme, but is homologous to a second subunit (LCB2) that is PLP-dependent [24].

(b) The products of genes AGXT2L1 and AGXT2L2 are homologs of the mitochondrial alanine-glyoxylate aminotransferase (AGXT2), but were recently shown to lack this activity [25].

(c) The murine homolog of this gene was shown to encode an enzyme with phospho-lyase activity [26].

(d) This gene is homolog to the plant 1-aminocyclopropane-1-carboxylate (ACC) synthase, but the gene product was shown to lack this activity [27].

(e) Genes ADC and AZIN1 encode ornithine decarboxylase antizyme inhibitors, i.e. proteins homologous to ornithine decarboxylase, but devoid of ornithine decarboxylase activity, that are involved in regulation of polyamine biosynthesis [28,29]. The human ADC, contrary to its mouse ortholog, retains a conserved lysine at the active site.

We also compared the functional classification provided by the B6 database with the manual annotation included in the NCBI 36 release of the human genome, finding no significant differences. This implies that the accuracy of our automatic classification system can match that of a manual expert annotation. It should be noted that only a minority of complete genomes have been subjected to accurate manual annotation. In genomes where proteins have been mostly annotated through a general system of automatic annotation, our specialized tool provides a more complete and accurate classification of PLP-dependent enzymes.

Of course, accuracy in the annotation of a gene product does not always guarantee a precise functional assignment, as it can be gleaned by inspecting Table 1. For example, some of the human PLP-dependent proteins in our inventory are homologs of enzymes (such as plant ACS synthases or bacterial threonine synthases) that are not expected to occur in mammals. In other cases, the proteins are homologs of other (functionally validated) human enzymes, but it is unclear whether they represent true isozymic forms, or rather possess distinct catalytic activities - this latter possibility may be especially pertinent for those sequences that were recognized as 'low-score' by our search procedure. These uncharacterized gene products represent therefore interesting subjects for functional genomic studies.

Some genes encoding for PLP-dependent enzymes may be missing from the list, possibly due to the limits of the current human genome assembly, even eight years after publication of the first genome draft [23]. For example, the gene ACCSL has been recognized as protein-coding only in the NCBI 36 assembly but was absent in the preceding version (NCBI 35).

## Conclusion

The increasing number of predicted protein sequences generated by genomic sequencing projects require methods to predict details regarding functions. The B6 database allows the comparison of newly sequenced PLP-dependent proteins with a curated collection of protein families, making it more reliable a preliminary functional classification but also helping to pinpoint gene products that are the most interesting candidates to functional studies.

Due to the progresses of functional genomics, as well as to classical biochemical and genetic approaches, the body of information on PLP-dependent enzymes is necessarily going to increase. Many activities that are currently 'orphan' (i.e., with no molecular details about the responsible enzymes) will be associated to specific sequences, while many new activities are likely to be discovered [6]. Accordingly, we expect to periodically update and expand the B6 database with the ensuing information, to maintain this database a serviceable tool and a reference point for the scientific community.

## Availability and requirements

The B6 database, which is based on the web-oriented Perl package Woda, is publicly available over the Internet . Users are asked to cite the present article.

## Abbreviations

PLP: pyridoxal 5'-phosphate; HMM: hidden Markov model; EC: enzyme Commission.

## Authors' contributions

RP designed and implemented the B6 database and website, carried out the genomic analysis and revised the manuscript. AP collected the literature included in the database, selected the functionally validated sequences, helped to build the families, drafted and revised the manuscript. Both authors read and approved the final manuscript.
